# The radiomics fingerprint of cartilage tumours: radiomics-based MRI differentiation of enchondroma and atypical cartilaginous tumour

**DOI:** 10.1007/s11604-026-01946-2

**Published:** 2026-02-05

**Authors:** Bökebatur Ahmet Raşit Mendi, Halitcan Batur, Nurdan Çay

**Affiliations:** 1 Department of Radiology, Faculty of Medicine, Ankara Yıldırım Beyazıt University, Üniversiteler Mah. İhsan Doğramacı Bulvarı Ankara Şehir Hastanesi Yanı Çankaya, 06800 Ankara, Turkey; 2 Department of Radiology, Falu Lasarett, 791 82 Falun, Sweden

**Keywords:** Enchondroma, Atypical cartilaginous tumour, MRI, Radiomics, Machine learning

## Abstract

**Purpose:**

This study aimed to develop and validate machine learning models based on quantitative radiomics parameters extracted from T1-weighted MRI to differentiate enchondromas from atypical cartilaginous tumours (ACTs).

**Methods:**

A retrospective cohort comprising 66 patients (35 with histopathologically confirmed enchondroma and 31 with ACT) was included in the study. T1-weighted MRI images were used for 2D segmentation, performed independently by two experienced observers on all visible slices of each lesion. A comprehensive set of 107 radiomics features was extracted from these segmented regions of interest. LASSO regression was applied for dimensionality reduction. Four distinct machine learning algorithms—Support Vector Machine (SVM), Random Forest Classifier (RFC), Extreme Gradient Boosting (XGBoost), and Decision Tree Analysis—were trained and validated using a 70:30 data split.

**Results:**

The radiomics features demonstrated high inter- and intra-observer reproducibility. All evaluated machine learning models exhibited strong diagnostic performance, with Area Under the Curve (AUC) values exceeding 0.90. Specifically, SVM achieved an AUC of 0.922 (95% CI 0.893–0.951), RFC yielded an AUC of 0.920 (95% CI 0.881–0.963), and Decision Tree Analysis showed an AUC of 0.949 (95% CI 0.927–0.972). Notably, the XGBoost model achieved the highest diagnostic efficacy, boasting an impressive AUC of 0.987 (95% CI 0.976–0.999), coupled with a sensitivity of 89.35% and a specificity of 96.55%.

**Conclusion:**

Our results indicate that the combination of MRI-based radiomics and machine learning algorithms, particularly XGBoost, offers a non-invasive and highly accurate method for distinguishing enchondroma from ACT.

**Supplementary Information:**

The online version contains supplementary material available at 10.1007/s11604-026-01946-2.

## Introduction

Cartilaginous bone tumours consist of chondroid matrix and tumour cells exhibiting chondroc3yte differentiation [1]. The majority of cartilaginous bone tumours are enchondromas, which are the second most frequent benign bone tumour [2]. The third most prevalent primary malignant tumour of the bone is chondrosarcoma, which is histologically classified into three grades [3].

The classification and management of these tumours have recently evolved. The 2020 World Health Organization (WHO) classification recommends the term 'Atypical Cartilaginous Tumour’ (ACT) for low-grade (Grade 1) chondroid lesions arising centrally within the long bones of the appendicular skeleton [4]. ACTs permeate and entrap pre-existing trabecular bone, which constitutes the primary histopathological distinction from enchondromas [4]. While enchondromas are typically managed with a watch-and-wait strategy, the management of ACTs has shifted towards conservative options such as active surveillance or intralesional curettage, often combined with an adjuvant therapy [5, 6]. In contrast, higher-grade chondrosarcomas (Grade 2 and 3) require extensive resection and are associated with a poorer prognosis [7].

Differentiating enchondroma from ACT remains a significant diagnostic challenge due to their overlapping clinical and radiological features [8]. In clinical practice, this differentiation frequently depends on a synthesis of imaging findings, lesion behaviour over time, and, where warranted, biopsy. However, even biopsy may yield inconclusive results, and diagnostic uncertainty can lead to either unnecessary surgery or delayed treatment [9, 10].

Magnetic Resonance Imaging (MRI) stands as the principal modality for evaluating cartilaginous tumours, offering superior soft-tissue contrast and multiplanar capabilities for the detailed assessment of intramedullary extension, cortical integrity, and soft-tissue components. [10]. Computed tomography (CT) may complement MRI by providing superior detection of matrix mineralization and subtle cortical disruption, but lacks sensitivity for marrow and soft tissue evaluation [11]. Despite these imaging techniques, differentiating enchondroma and ACT remains a significant challenge. Both lesions frequently exhibit overlapping radiological features, such as lobulated contours, hyperintense T2 signals, intralesional calcifications and endosteal scalloping, which can lead to diagnostic ambiguity. [12]. Features like endosteal scalloping and mild cortical thinning, while often observed in benign enchondromas—especially in long bones—can also be seen in low-grade chondrosarcomas, making the radiological discrimination difficult [13]. The convergence of these findings frequently leads to diagnostic ambiguity, underscoring the need for more objective and quantitative assessment tools.

Radiomics, an emerging field in quantitative imaging, enables the extraction of a large number of quantitative features from conventional medical images, such as MRI, that are imperceptible to the human eye. These features reflect tumour shape, texture, intensity, and heterogeneity, offering a more objective and reproducible characterization of tissue [14]. First-order radiomics parameters reflect the intensity distribution within the segmented image, whereas second-order parameters reveal the statistical correlations between voxels in three-dimensional space [15]. In recent years, radiomics-based approaches have shown promising results in differentiating various musculoskeletal tumours [16, 17].

This study aimed to develop machine learning models based on radiomics features extracted from T1-weighted MRI to discriminate enchondroma from ACT. Radiomics features were obtained from histopathologically confirmed lesions and employed to train and validate supervised classifiers. We hypothesize that even T1-weighted MRI alone can provide sufficient quantitative information for accurate, non-invasive lesion classification.

## Material and methods

### Study population

This retrospective study was approved by the institutional review board (IRB) of Ankara City Hospital (decision number: E2-21-911) The IRB waived the necessity of consent due to the retrospective design, anonymised data gathering, and the absence of risk to participating individuals.

**Fig. 1:**
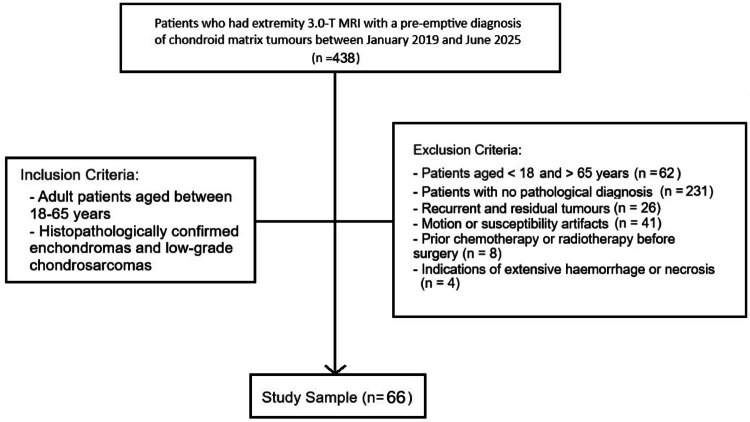
Figure illustrating patient selecting process

**Fig. 2:**
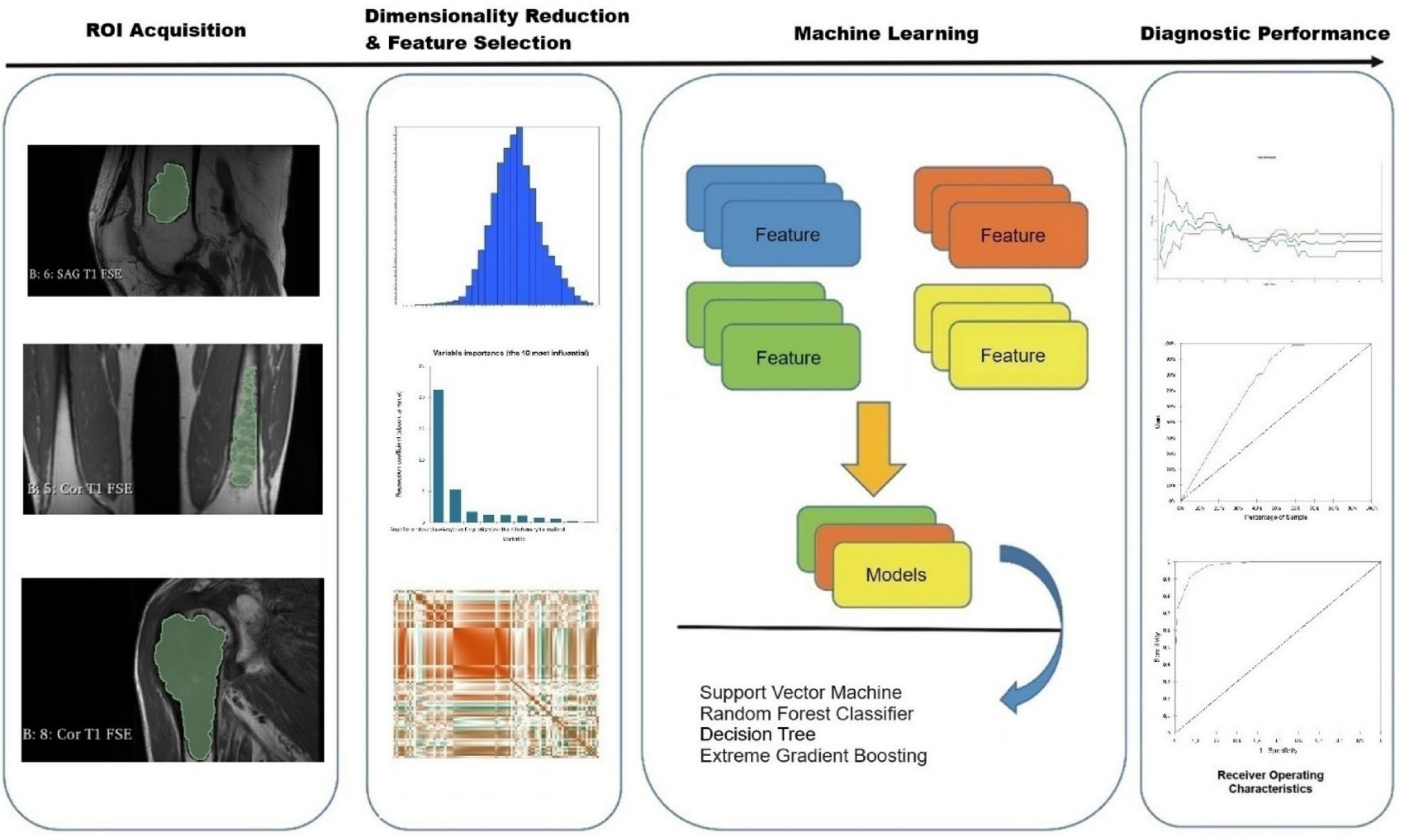
Figure depicting flowchart of study

The institutional database was scanned for patients who had undergone preoperative extremity 3.0-T MRI with a pre-emptive diagnosis of chondroid matrix tumours between January 2019 and June 2025. The exclusion criteria encompassed recurrent or residual tumours, motion or susceptibility artifacts that hindered evaluation and segmentation process, prior chemotherapy or radiotherapy before surgery, a pathological diagnosis other than enchondroma or ACT, and indications of extensive haemorrhage or necrosis. Individuals of both genders aged 18 to 65 who met the criteria were included into the sample. Histopathology reports were used to classify the cases into the enchondroma and the ACT group. All cases in our sample were histopathologically confirmed following surgical removal of long bone lesions that had been resected due to radiologic and clinical uncertainty. The histopathological diagnosis was established by experienced musculoskeletal pathologists, who distinguished ACT from enchondroma based on key histological features such as increased cellularity, mild nuclear atypia, and most importantly, a permeative growth pattern with entrapment of host trabecular bone, in accordance with WHO guidelines. In controversial cases, the final diagnosis was documented by consensus of the pathologists in our institution. Patient selection is summarized in Fig. 1 and the flowchart of the study is depicted in Fig. 2.

### MRI acquisition and pre-processing

The images were acquired via a 3-T scanner (Signa Pioneer, GE Healthcare, Milwaukee, USA). T1-weighted fast spin echo (FSE) images in the coronal and sagittal planes were selected for analysis, as they were the most consistently available sequence across all anatomical locations in our retrospective dataset, allowing for a uniform comparison. The parameters for 2D T1-weighted images were as follows: slice thickness = 2.5 mm; slice gap = 0.3 mm; pixel size = 0.43 × 0.43 mm2; repetition time = 630 ms; echo time = 10 ms and echo train length = 3.

**Fig. 3:**
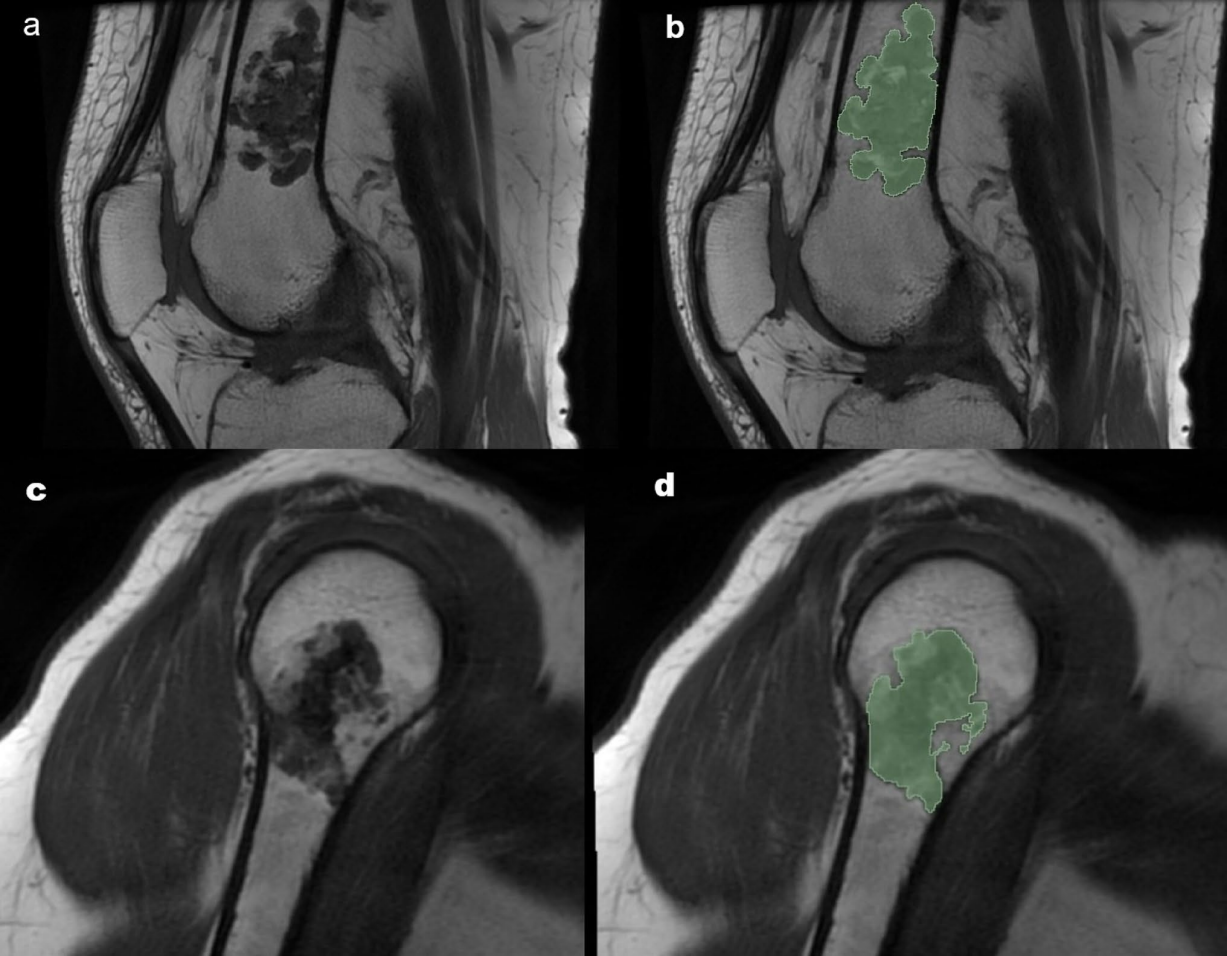
Segmentation process for two representative cases: an ACT and an enchondroma. A sagittal T1-weighted image (**a**) and corresponding segmentation (**b**) show a lesion in the diaphysis of the distal femur, histopathologically confirmed as ACT. A sagittal T1-weighted image (**c**) and corresponding segmentation (**d**) demonstrate a lesion in the proximal metaphysodiaphyseal region of the humerus, confirmed as enchondroma

Relevant images were uploaded to 3D Slicer software version 5.6.2 [18] and 2D segmentations were performed by two independent observers (HB and BARM, with nine and seven years of experience, respectively). Each lesion was delineated in all slices included in the MRI examination. In each section, the margins of the ROI containing the tumour were determined manually using the free-hand method. All areas where the lesion did not extend, including adjacent cortical bone, muscle, and vessels, were meticulously excluded in each segment while selecting the borders. Examples of the segmentation process is presented as Fig. 3.

### Acquisition of radiomics parameters

Radiomics analysis was performed post-segmentation employing the SlicerRadiomics module, which employs the PyRadiomics library [19]. Voxels were resampled to 1 × 1 × 1 mm^3^. For gray-level discretization, a fixed bin width of 64 was used. The relationship between two adjacent voxels, which forms the basis for the calculation of second-order parameters, was established using offsets of 1 voxel and 13 displacement vectors in different directions.

The acquisition of 2D ROIs limited the use of certain shape and size-based features that necessitate absolute three-dimensional volumes of interest (VOIs), such as sphericity, compactness, smoothness, and volume, for radiomics analysis. Following the establishment of the presets, 107 radiomics parameters were calculated and classified as shape, first-order, Gray Level Co-occurrence Matrix (GLCM), Gray Level Run Length Matrix (GLRLM), Neighbouring Gray-scale Difference Matrix (NGTDM), Gray Level Size Zone (GLSZM), and Gray Level Dependence Matrix (GLDM).

### Statistical analysis

The Kolmogorov–Smirnov test was used to determine the normality of distribution. Normally distributed data were given as mean ± SD, while non-normally distributed data were presented as median and interquartile range.

Spearman’s Rank correlation was used to examine inter-observer reproducibility of chosen parameters. A cutoff of P ≥ 0.8 was used to assess reproducibility for each parameter.

Intra-observer reproducibility was evaluated using intraclass correlation coefficient (ICC). Reproducibility was defined as an ICC value of ≥ 0.75 [20].

Dimensionality reduction was performed using the Least Absolute Shrinkage and Selection Operator (LASSO) on the training set. A tenfold cross-validation was performed to identify the optimum lambda regularization parameter, and the association between mean standard error (MSE) and lambda was given. The significance of parameters was determined using regression coefficients. The XLStat statistical tool, version 2024.2.2 (Addinsoft, NY, USA), was utilized for analyses. Statistical significance was defined as a P-value < 0.05 for all analyses.

### Machine learning

Random Forest Classifier (RFC) analysis [21], Support Vector Machine (SVM) [22], Extreme Gradient Boosting (XGBoost) [23], and Decision Tree Analysis [24] were used as machine learning methods. The study population was randomly partitioned into training and validation sets in a 70:30 ratio for validation purposes. The sensitivity, specificity, and area under the curve (AUC) values of all methods were provided.

The SVM parameters were configured as follows: linear kernel; C value = 1.0; tolerance = 0.001. Due to the extensive variability of radiomics parameter values, standardization was implemented as a preprocessing step. To mitigate variability and selection bias in performance estimations, tenfold bootstrapping was performed, adhering to the methodology suggested by Vrigazova et al. [25].

The RFC settings were determined as follows: bagging method; random sampling with replacement; number of trees = 200; and maximum depth = 20. Alongside the aforementioned data, the misclassification rate, out-of-bag error evolution chart, and mean decrease in accuracy (indicating parameter significance) were also documented.

The parameters for XGBoost were configured as follows: maximum iterations = 100; learning rate = 0.3; minimum loss reduction = 0; objective function = quadratic; and maximum tree depth = 6. Alongside the aforementioned metrics, gain and lift curves were also presented.

The parameters for the Decision Tree Analysis were as follows: tree type = classification; method = chi-square automatic interaction detection (CHAID); maximum tree depth = 3; significance level = 0.05; merge threshold = 0.05; and twofold cross-validation. The aforementioned parameters were given together with lift and gain curves.

## Results

### Study population

The study included 66 patients, consisting of 35 individuals with enchondroma and 31 with ACT. The long bones of the appendicular skeleton were the site of all lesions, with 22 enchondromas in the lower extremities and 13 in the upper extremities. Eight of the 31 ACTs were located in the upper extremities, while twenty-three were in the lower extremities. The average of the largest diameter of lesions measured on sagittal and coronal sections was 38 ± 8 mm in enchondromas and 44 ± 7 mm in ACTs. The difference in location (p = 0.435) and size (p = 0.123) between the groups were not statistically significant.

The mean age in the enchondroma group was 43.1 ± 13.4 years (range: 21–58), whereas the ACT group had a mean age of 46.2 ± 14.1 years (range: 23–65). The age disparity between the two groups was not statistically significant (p = 0.24). The enchondroma group comprised 14 females and 21 males, while the ACT group consisted of 17 females and 14 males. The difference was not statistically significant (p = 0.14).

Pain was reported in 12 of the 35 patients with enchondroma (34%), whereas 18 of the 31 patients with ACT (58%) presented with pain. Although the frequency of pain appeared higher in the ACT group, this difference was not statistically significant (p = 0.091). All findings are summarized in Table 1.

**Table 1:** Comparison of demographic and clinical characteristics between patients with enchondroma and atypical cartilaginous tumors (ACT)

Characteristic	Enchondroma (n = 35)	ACT (n = 31)	p-value
**Age (years, mean ± SD)**	**43.1 ± 13.4**	**46.2 ± 14.1**	**0.24**
**Sex (Female / Male)**	**14 / 21**	**17 / 14**	**0.14**
**Location**			**0.435**
*Upper Extremity*	13 (37%)	8 (26%)	
Humerus	7	4	
Radius	3	2	
Ulna	3	2	
*Lower Extremity*	22 (63%)	23 (74%)	
Femur	12	13	
Tibia	8	9	
Fibula	2	1	
**Presence of Pain**	**12 (34%)**	**18 (58%)**	**0.091**
**Size (mm, mean ± SD)**	**38 ± 8**	**44 ± 7**	**0.123**

The bold font is used to denote primary characteristics, whereas the plain font indicates their respective sub-classifications

### Inter- and intra-observer reproducibility

Ten patients who provided a total of 53 ROIs per observer during the segmentation procedure were selected randomly. Then, “mean” and “range” radiomics parameters from the first order group were used to measure interobserver reproducibility. Both parameters indicated significant correlation between the two observers based on Spearman’s rank coefficients (mean: ρ = 0.822, P = 0.018; range: ρ = 0.841, P = 0.027).

The same randomly selected patients were utilized to measure intra-observer reproducibility. Two observers conducted the segmentation process twice. Then, “mean” and “range” parameters were used. Based on both parameters, intra-observer agreement was found to be good for each observer. For Observer 1, the mean intraclass correlation coefficient (ICC) was 0.821, with a range of 0.738 to 0.902. Observer 2 showed a mean ICC of 0.854, ranging from 0.794 to 0.918.

### Dimensionality reduction of the parameters

LASSO regression identified ten parameters that exhibited minimal correlation and significant discriminative power. The lambda value that was found to be optimal was 0.014. Supplementary Fig. 1 depicts the correlation matrix, while Table 2 displays the most significant parameters and their standardized coefficients. Supplementary Fig. 2 illustrates the correlation between the lambda parameter and the mean standard error (MSE). Table 3 depicts the group mean comparisons of the ten most significant parameters.

**Table 2:** The most significant radiomics parameters based on LASSO regression

Parameter	Standardized Coefficient
SmallDependenceLowGrayLevelEmphasis	− 0.093
ShortRunLowGrayLevelEmphasis	− 0.326
Idn	0.072
DependenceNonuniformityNormalized	0.18
SizeZoneNonunifortmityNormalized	− 0.24
SmallAreaEmphasis	− 0.042
RunPercentage	− 0.044
InterquartileRange	− 0.058
Coarseness	− 0.133
Energy	0.115

LASSO, least absolute shrinkage and selection operator

**Table 3:** Group mean comparisons of the ten most significant parameters

Parameter	Enchondroma group mean ± SD	ACT group mean ± SD	*P* Value
Coarseness	8,273,262,300.56 ± 48,589,309,440.78	2,738,694,542.78 ± 10,714,237,666.53	< 0.001
Small Dependence Low Gray Level Emphasis	0.00366 ± 0.00287	0.00135 ± 0.00125	< 0.001
Energy	14,121,623,115.21 ± 10,885,839,870.85	60,281,547,291.45 ± 19,905,322,246.13	< 0.001
Idn	0.9126 ± 0.0193	0.9615 ± 0.0221	< 0.001
Small Area Emphasis	0.5616 ± 0.0636	0.4406 ± 0.0561	< 0.001
Size Zone NonUniformity Normalized	0.3067 ± 0.0493	0.2377 ± 0.0309	0.001
RunPercentage	0.8546 ± 0.1239	0.6641 ± 0.1013	0.002
Dependence NonUniformity Normalized	0.1979 ± 0.0509	0.1033 ± 0.0448	0.002
Interquartile Range	377.01 ± 21.19	337.17 ± 19.20	0.022
Short Run Low Gray Level Emphasis	0.2185 ± 0.0321	0.1740 ± 0.0146	0.041

### Diagnostic performance of ML algorithms

The diagnostic performance of SVM was as follows: sensitivity = 90.5% (95% confidence interval [CI] = 87.33–93.66); specificity = 87.25% (95% CI = 83.73– 90.15); and AUC = 0.922 (95% CI = 0.893–0.951).

**Fig. 4:**
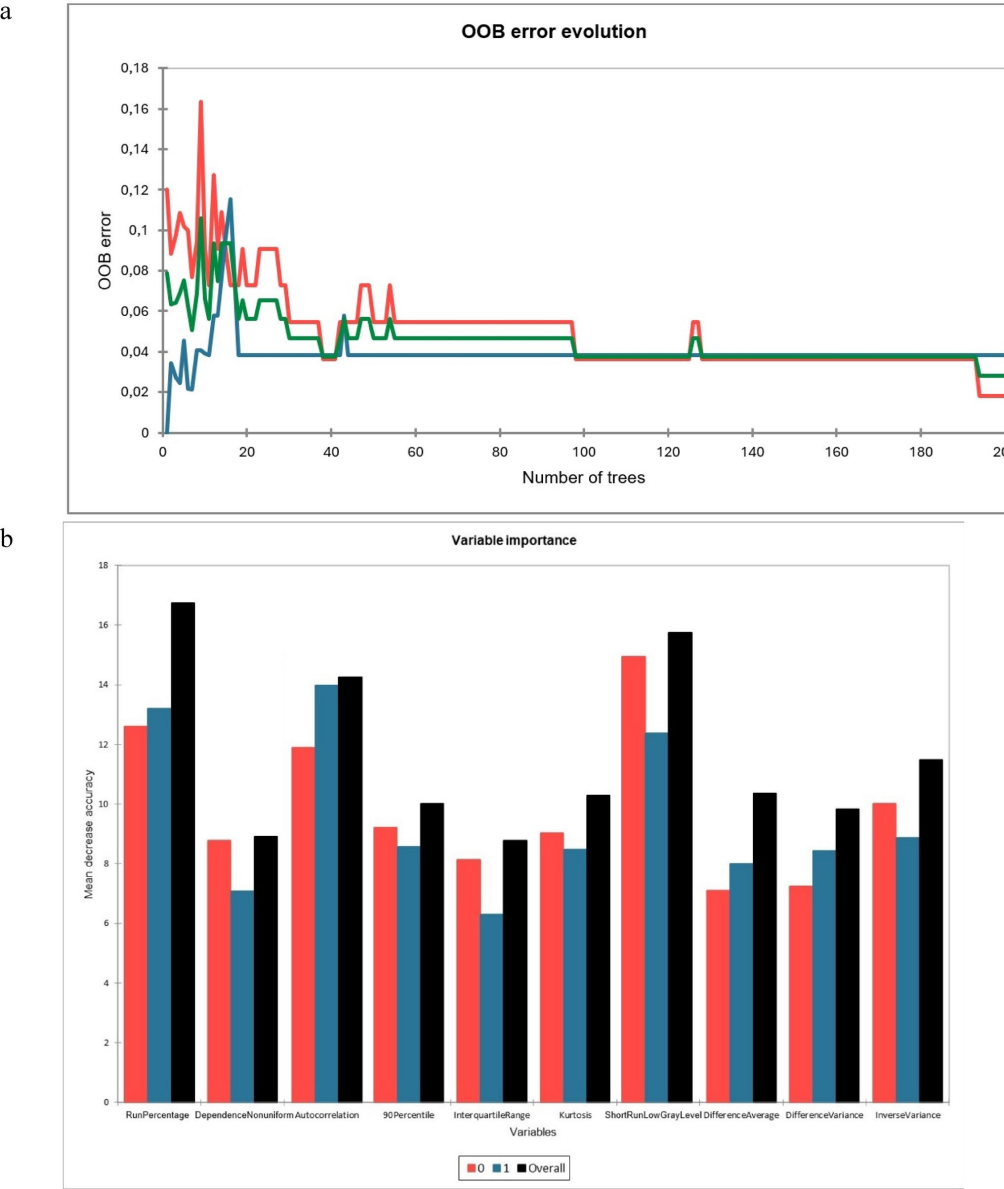
The out-of-bag (OOB) error evolution (**a**) chart of Random Forest Classifier. X axis: Number of trees built; Y axis: Error rate. Red line represents error rate for “low-grade chondrosarcoma” diagnosis. Blue line represents error rate for “enchondroma” diagnosis. Green line represents out-of-bag observations. Mean decrease accuracy chart (**b**) depicts variable importances

The diagnostic efficiency of RFC was as follows: misclassification rate = 0.08; sensitivity = 88.37% (95% CI = 85.21–91.31); specificity = 87.32% (95% CI = 83.73–90.64); and AUC = 0.92 (95% CI = 0.881–0.963). Figure 4 displays the mean decreasing accuracy and out-of-bag (OOB) error evolution charts.

**Fig. 5:**
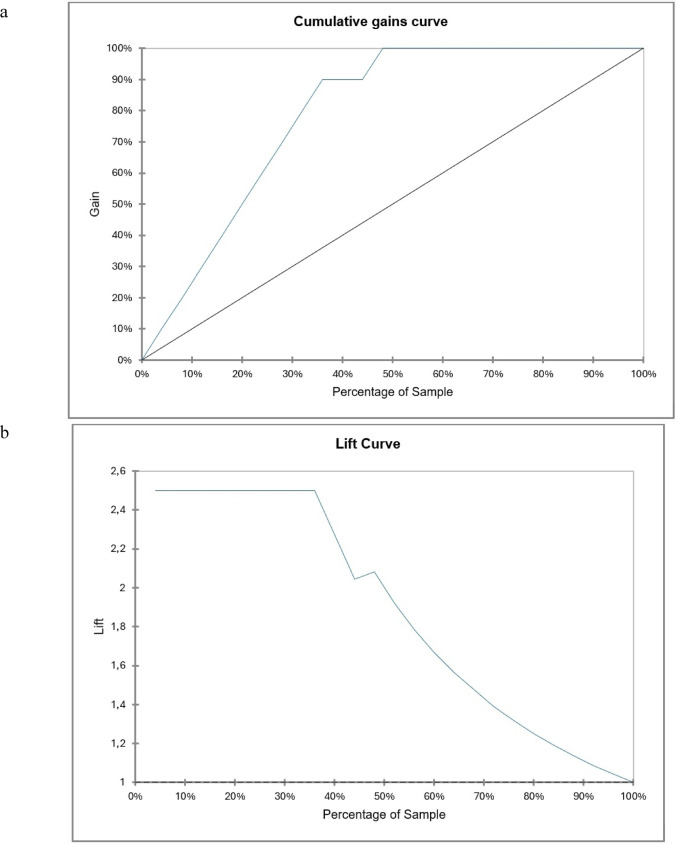
The diagnostic performance of XGBoost was illustrated with gain (**a**) and lift (**b**) curves. The Gain curve plots the true positive rate in percent versus the percent of total observations. Lift is the ratio of the model’s gain to the percentage of the dataset utilized

The diagnostic efficacy of XGBoost was as follows: sensitivity = 89.35% (95% CI = 85.72–92.89); specificity = 96.55% (95% CI = 93.5–99.7); and AUC = 0.987 (95% CI = 0.976–0.999). The gain and lift curves are depicted in Fig. 5.

**Fig. 6:**
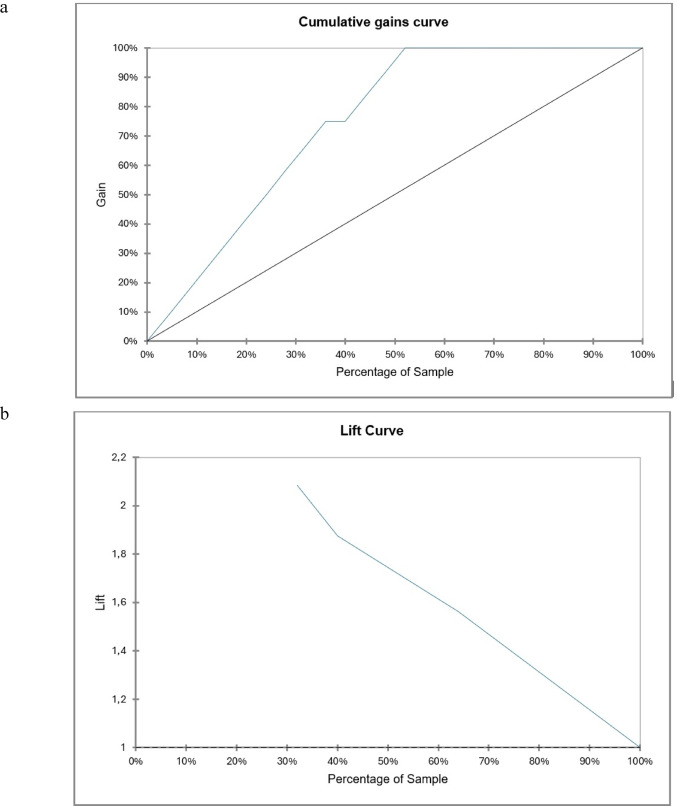
The diagnostic effectiveness of Decision Tree was illustrated with gain (**a**) and lift (**b**) curves. The Gain curve plots the true positive rate in percent versus the percent of total observations. Lift is the ratio of the model’s gain to the percentage of the dataset utilized

The diagnostic performance of Decision Tree Analysis was as follows: sensitivity = 92.44% (95% CI = 88.61– 96.05); specificity = 91.75% (95% CI = 88.05–95.56); and AUC = 0.949 (95% CI = 0.927–0.972). The gain and lift curves are illustrated in Fig. 6.

## Discussion

In order to distinguish enchondromas from ACTs using MRI, this study sought to investigate the capabilities of combining radiomics with machine learning techniques. Our findings suggest that the analysis of quantitative radiomics parameters using machine learning algorithms offers a promising potential to improve diagnostic accuracy, even though it can be challenging to distinguish between these cartilage tumours using conventional imaging alone. Remarkably, the approach we employed produced AUC values higher than 0.90, with the XGBoost model obtaining a noteworthy AUC of 0.987. This robust performance underscores the potential of our non-invasive method to offer valuable insights in situations where traditional assessment may be ambiguous, thereby assisting clinicians in making more informed decisions about patient management.

The distinction between enchondroma and ACT poses a significant challenge for radiologists, pathologists, and clinicians in clinical practice, despite numerous attempts in the literature to overcome this difficulty [26]. Conventional radiological methods have shown limited success in this differentiation, primarily due to high rates of both false-positive and false-negative diagnoses [7]. A 2012 study by Ferrer-Santacreu et al. investigating the role of MRI in differentiating the two entities in the appendicular skeleton found no statistically significant distinguishing MRI features [27]. These findings were contradicted a year later by Choi et al., who identified several MRI features suggestive of malignancy, including a predominantly intermediate signal intensity matrix on T1-weighted imaging, a multilobulated enhancement pattern, cortical destruction, an associated soft tissue component, surrounding bone marrow and/or soft tissue oedema, epiphyseal involvement, and/or location in a flat bone [10]. In 2015, Crim et al. assessed the efficacy of MRI and radiography in distinguishing enchondroma from low-grade chondrosarcoma in 53 patients. In assessing low-grade lesions, they discovered that both radiographs and MRI had limitations, and bone expansion and cortical thickening were uncommon but distinct signs of malignancy [7]. Moreover, it has been reported that there is considerable overlap in the enhancement patterns of enchondromas and low-grade chondrosarcomas on dynamic contrast-enhanced MRI, limiting its value in differentiation [9]. This highlights the controversy in the literature, as other studies, such as the one by De Coninck et al., have suggested it to be a useful tool [28]. Discrimination by conventional strategies continues to be considered a diagnostic challenge. As of right now, no documented standardized management guidelines have been published.

Recent advancements in radiomics and machine learning (ML) offer promising avenues for differentiating enchondroma from chondrosarcoma. Gitto et al. (2021) investigated the influence of interobserver variability on 2D and 3D CT- and MRI-based texture feature reproducibility specifically in cartilaginous bone tumours, highlighting that radiomics features extracted from unenhanced CT and MRI are reproducible, despite some interobserver segmentation variability [29]. Pan et al. (2021) further contributed by developing radiomics nomograms based on non-enhanced MRI and clinical risk factors (tumour location, age, sex), achieving high AUCs (up to 0.961 in the validation group) for differentiating chondrosarcoma from enchondroma, particularly emphasizing the strong performance of T1-weighted and combined T1-weighted + T2-weighted features [30]. Erdem et al. (2023) demonstrated that neural networks, among seven ML models, achieved high accuracy (AUC 0.979–0.990) in distinguishing these lesions using radiomics features from T1 and PD-weighted MRI [31]. Cilengir et al. (2023) reported promising diagnostic value for MRI-based texture analysis, with the K-neighbours classifier achieving an AUC of 1.00 for FS-PD images in differentiating enchondroma and chondrosarcoma [32]. Although the systematic review conducted by Zhong et al. (2023) concluded that the quality of radiomics studies in chondrosarcoma was insufficient (median Radiomics Quality Score: 10.5), it also noticed the potential for optimising clinical decision-making [33].

To our knowledge, this study is the first to specifically target the differentiation of enchondroma and ACT using radiomics and machine learning on MRI examinations. Although literature on radiomics research aimed at distinguishing enchondroma from low-grade chondrosarcoma is limited, different modalities aside from MRI have been studied. For instance, Yoon et al. investigated the value of SPECT/CT radiomics parameters in differentiating enchondroma and grade I chondrosarcomas in long bones [34]. Their study, involving 49 patients, found that a specific radiomics parameter (zone-length non-uniformity) was a significant independent factor for distinction, achieving a sensitivity of 83.3% and specificity of 90.9% in their test data. Nevertheless, their study was limited by the absence of machine learning algorithms and a relatively small sample size. Similarly, Yildirim et al. developed a CT radiomics-based machine learning model to differentiate low-grade chondrosarcoma from enchondroma [35]. This study, with 30 enchondroma and 26 chondrosarcoma patients, utilized 3D CT-based radiomics and various machine learning models. They reported that a random forest model using 5 parameters achieved an AUC of 0.967. A limitation of this study was also its retrospective nature, as well as the relatively small cohort size.

Our study’s focus on T1-weighted MRI further highlights its potential for widespread clinical applicability, given the common use and consistency of this sequence across various anatomical locations in musculoskeletal imaging. Radiomics approach employed in our study extracted 107 quantitative parameters from T1-weighted MRI images, which were subsequently reduced to 10 most discriminative parameters using LASSO regression. This dimensionality reduction technique helped identify the most relevant features while avoiding overfitting, a common challenge in radiomics studies. XGBoost (AUC = 0.987) performed better than other machine learning methods like SVM (0.922), Decision Tree (0.949), and even Random Forest (RFC: 0.92). This is most likely attributable to its gradient boosting approach. XGBoost builds a strong prediction model by combining many simple models, usually decision trees, one after another. This step-by-step improvement helps XGBoost find complex patterns in detailed medical image data, often doing better than single models or other ensemble methods like Random Forest. Its strength comes from L1 and L2 regularization techniques that prevent overfitting and make it more generalizable, which is key for medical data with limited samples [23]. The high specificity (96.55%) achieved by XGBoost is particularly noteworthy, as it suggests a low false-positive rate, which is crucial in clinical practice to avoid unnecessary surgical interventions for enchondromas.

The performance of our T1-based radiomics model may be explained by its ability to quantify subtle, pixel-level textural variations that are often imperceptible to the human eye, yet appear to correspond to key histopathological differences. ACTs are defined by features such as increased cellularity and a permeative growth pattern, which can disrupt the uniform signal of surrounding marrow fat and create a more complex, heterogeneous texture on T1-weighted images. An analysis of the ten most discriminative features selected by LASSO regression, detailed in Table 2, lends support to this hypothesis. For instance, a higher DependenceNonuniformityNormalized (coefficient: 0.180) was a strong predictor for ACT, suggesting that textural heterogeneity is a key indicator of malignancy. This is further supported by the Coarseness parameter (coefficient: -0.133); a lower coarseness value, which points to a finer and less uniform texture, was also associated with ACT. This could reflect the microscopic architectural disruption caused by permeative growth. Conversely, a high ShortRunLowGrayLevelEmphasis (coefficient: -0.326) was the strongest predictor for enchondroma, potentially reflecting its more organized and less aggressive structure. In essence, our model seems to move beyond conventional radiological assessment by translating these microscopic, visually elusive hallmarks of malignancy into a quantitative signature.

Our research possesses a few limitations. First, the study was a single-centre retrospective research with a relatively limited sample size. Multi-centre prospective studies with larger samples are needed to validate our findings and evaluate the generalizability of our radiomics models. Second, our analysis was limited to T1-weighted images and 2D segmentations. The inclusion of other MRI sequences (such as T2-weighted, STIR, or contrast-enhanced sequences) with the acquisition of 3D volume of interests (VOIs) might further improve diagnostic capability. Additionally, the development of automated segmentation could further streamline the clinical implementation of radiomics-based diagnosis. We also acknowledge that we did not systematically analyze intratumoural mineralization, a potential confounder given its effect on MRI signal. However, the textural heterogeneity caused by mineralization may also represent a valuable source of data for our radiomics parameters. The high accuracy achieved by the radiomics-based ML algorithms suggests that these complex patterns, while challenging for visual interpretation, may contain valuable information for quantitative classification.

In conclusion, our study demonstrates that MRI-based radiomics combined with machine learning, particularly XGBoost, can achieve robust diagnostic performance in differentiating enchondroma from ACT. While this non-invasive approach has the potential to significantly improve diagnostic accuracy and clinical decision-making, its findings must be validated by prospective studies with larger sample sizes.

## Supplementary Information

Below is the link to the electronic supplementary material.

Supplementary Figure 1: The correlation matrix of Least Absolute Shrinkage And SelectionOperator (LASSO). Supplementary Material 1

Supplementary Figure 2: Correlation of mean standard error (MSE) with lambda parameter. Supplementary Material 2

## References

[1] Jo VY, Fletcher CD. WHO classification of soft tissue tumours: an update based on the 2013 (4th) edition. Pathology. 2014;46(2):95–104. 24378391 10.1097/PAT.0000000000000050

[2] Stomp W, Reijnierse M, Kloppenburg M, de Mutsert R, Bovée JV, den Heijer M, et al. Prevalence of cartilaginous tumours as an incidental finding on MRI of the knee. Eur Radiol. 2015;25(12):3480–7. 25994192 10.1007/s00330-015-3764-6 PMC4636526

[3] Murphey MD, Walker EA, Wilson AJ, Kransdorf MJ, Temple HT, Gannon FH. From the archives of the AFIP: imaging of primary chondrosarcoma: radiologic-pathologic correlation. Radiographics. 2003;23(5):1245–78. 12975513 10.1148/rg.235035134

[4] Anderson WJ, Doyle LA. Updates from the 2020 World Health Organization classification of soft tissue and bone tumours. Histopathology. 2021;78(5):644–57. 33438273 10.1111/his.14265

[5] Leithner A, Smolle MA. The Enigma of Atypical Cartilaginous Tumors: Surgery or Surveillance? : MDPI; 2023. p. 4696. 10.3390/cancers15194696 PMC10571963 37835390

[6] Scholte CH, de Van San MA, der Van Wal RJ, Broekhuis D, Van Langevelde K, Dorleijn DM. Clinical outcome of curettage in atypical cartilaginous tumors of the long bones: a descriptive cohort study. Acta Orthop. 2024;95:752. 39713913 10.2340/17453674.2024.42636 PMC11664434

[7] Crim J, Schmidt R, Layfield L, Hanrahan C, Manaster BJ. Can imaging criteria distinguish enchondroma from grade 1 chondrosarcoma? Eur J Radiol. 2015;84(11):2222–30. 26220916 10.1016/j.ejrad.2015.06.033

[8] Wang X, De Beuckeleer L, De Schepper A, Van Marck E. Low-grade chondrosarcoma vs enchondroma: challenges in diagnosis and management. Eur Radiol. 2001;11(6):1054–7. 11419152 10.1007/s003300000651

[9] Douis H, Parry M, Vaiyapuri S, Davies A. What are the differentiating clinical and MRI-features of enchondromas from low-grade chondrosarcomas? Eur Radiol. 2018;28(1):398–409. 28695356 10.1007/s00330-017-4947-0

[10] Choi B-B, Jee W-H, Sunwoo H-J, Cho J-H, Kim J-Y, Chun K-A, et al. MR differentiation of low-grade chondrosarcoma from enchondroma. Clin Imaging. 2013;37(3):542–7. 23041161 10.1016/j.clinimag.2012.08.006

[11] Flemming DJ, Murphey MD. Enchondroma and chondrosarcoma. Semin Musculoskelet Radiol. 2000;4(1):59–71. 11061692 10.1055/s-2000-6855

[12] Lisson CS, Lisson CG, Flosdorf K, Mayer-Steinacker R, Schultheiss M, von Baer A, et al. Diagnostic value of MRI-based 3D texture analysis for tissue characterisation and discrimination of low-grade chondrosarcoma from enchondroma: a pilot study. Eur Radiol. 2018;28(2):468–77. 28884356 10.1007/s00330-017-5014-6

[13] Bui KL, Ilaslan H, Bauer TW, Lietman SA, Joyce MJ, Sundaram M. Cortical scalloping and cortical penetration by small eccentric chondroid lesions in the long tubular bones: not a sign of malignancy? Skeletal Radiol. 2009;38(8):791–6. 19277645 10.1007/s00256-009-0675-0

[14] Varghese BA, Cen SY, Hwang DH, Duddalwar VA. Texture analysis of imaging: what radiologists need to know. AJR Am J Roentgenol. 2019;212(3):520–8. 30645163 10.2214/AJR.18.20624

[15] Koçak B, Durmaz EŞ, Ateş E, Kılıçkesmez Ö. Radiomics with artificial intelligence: a practical guide for beginners. Diagn Interv Radiol. 2019;25(6):485. 31650960 10.5152/dir.2019.19321 PMC6837295

[16] Batur H, Mendi BAR, Cay N. Bone marrow lesions of the femoral head: can radiomics distinguish whether it is reversible? Pol J Radiol. 2023;88:e194. 37234462 10.5114/pjr.2023.127055 PMC10207319

[17] Cay N, Mendi BAR, Batur H, Erdogan F. Discrimination of lipoma from atypical lipomatous tumor/well-differentiated liposarcoma using magnetic resonance imaging radiomics combined with machine learning. Jpn J Radiol. 2022;40(9):951–60. 35430677 10.1007/s11604-022-01278-x

[18] Fedorov A, Beichel R, Kalpathy-Cramer J, Finet J, Fillion-Robin J-C, Pujol S, et al. 3D slicer as an image computing platform for the quantitative imaging network. Magn Reson Imaging. 2012;30(9):1323–41. 22770690 10.1016/j.mri.2012.05.001 PMC3466397

[19] Van Griethuysen JJ, Fedorov A, Parmar C, Hosny A, Aucoin N, Narayan V, et al. Computational radiomics system to decode the radiographic phenotype. Can Res. 2017;77(21):e104–7. 10.1158/0008-5472.CAN-17-0339 PMC5672828 29092951

[20] Koo TK, Li MY. A guideline of selecting and reporting intraclass correlation coefficients for reliability research. J Chiropr Med. 2016;15(2):155–63. 27330520 10.1016/j.jcm.2016.02.012 PMC4913118

[21] Breiman L. Random forests. Mach Learn. 2001;45(1):5–32.

[22] Borstelmann SM. Machine learning principles for radiology investigators. Acad Radiol. 2020;27(1):13–25. 31818379 10.1016/j.acra.2019.07.030

[23] Chen T, Guestrin C, editors. Xgboost: A scalable tree boosting system. In: Proceedings of the 22nd acm sigkdd international conference on knowledge discovery and data mining; 2016.

[24] Navada A, Ansari AN, Patil S, Sonkamble BA, editors. Overview of use of decision tree algorithms in machine learning. 2011 IEEE control and system graduate research colloquium; 2011. IEEE.

[25] Vrigazova B, Ivanov I. Tenfold bootstrap procedure for support vector machines. Computer Science. 2020;21:241–57.

[26] Afonso PD, Isaac A, Villagrán JM, editors. Chondroid tumors as incidental findings and differential diagnosis between enchondromas and low-grade chondrosarcomas. Seminars in musculoskeletal radiology; 2019: Thieme Medical Publishers. 10.1055/s-0038-1675550 30699449

[27] Ferrer-Santacreu EM, Ortiz-Cruz EJ, González-López JM, Pérez Fernández E. Enchondroma versus low‐grade chondrosarcoma in appendicular skeleton: clinical and radiological criteria. J Oncol. 2012;2012(1):437958. 22593766 10.1155/2012/437958 PMC3346996

[28] De Coninck T, Jans L, Sys G, Huysse W, Verstraeten T, Forsyth R, et al. Dynamic contrast-enhanced MR imaging for differentiation between enchondroma and chondrosarcoma. Eur Radiol. 2013;23(11):3140–52. 23771600 10.1007/s00330-013-2913-z

[29] Gitto S, Cuocolo R, Emili I, Tofanelli L, Chianca V, Albano D, et al. Effects of interobserver variability on 2D and 3D CT-and MRI-based texture feature reproducibility of cartilaginous bone tumors. J Digit Imaging. 2021;34(4):820–32. 34405298 10.1007/s10278-021-00498-3 PMC8455795

[30] Pan J, Zhang K, Le H, Jiang Y, Li W, Geng Y, et al. Radiomics nomograms based on non‐enhanced MRI and clinical risk factors for the differentiation of chondrosarcoma from enchondroma. J Magn Reson Imaging. 2021;54(4):1314–23. 33949727 10.1002/jmri.27690

[31] Erdem F, Tamsel İ, Demirpolat G. The use of radiomics and machine learning for the differentiation of chondrosarcoma from enchondroma. J Clin Ultrasound. 2023;51(6):1027–35. 37009697 10.1002/jcu.23461

[32] Cilengir AH, Evrimler S, Serel TA, Uluc E, Tosun O. The diagnostic value of magnetic resonance imaging-based texture analysis in differentiating enchondroma and chondrosarcoma. Skeletal Radiol. 2023;52(5):1039–49. 36434265 10.1007/s00256-022-04242-y

[33] Zhong J, Hu Y, Ge X, Xing Y, Ding D, Zhang G, et al. A systematic review of radiomics in chondrosarcoma: assessment of study quality and clinical value needs handy tools. Eur Radiol. 2023;33(2):1433–44. 36018355 10.1007/s00330-022-09060-3

[34] Yoon H, Choi WH, Joo MW, Ha S, Chung Y-A. SPECT/CT radiomics for differentiating between enchondroma and grade I chondrosarcoma. Tomography. 2023;9(5):1868–75. 37888740 10.3390/tomography9050148 PMC10610631

[35] Yildirim M, Yildirim H. CT radiomics-based machine learning model for differentiating between enchondroma and low-grade chondrosarcoma. Med. 2024;103(33):e39311. 10.1097/MD.0000000000039311 PMC11332721 39151512

