# Supplementary figures and images for: The radiomics fingerprint of cartilage tumours: radiomics-based MRI differentiation of enchondroma and atypical cartilaginous tumour
Source: Jpn J Radiol. 2026 Feb 5;44(6):1050–61. doi: 10.1007/s11604-026-01946-2 (PMC13222190; doi:10.1007/s11604-026-01946-2)

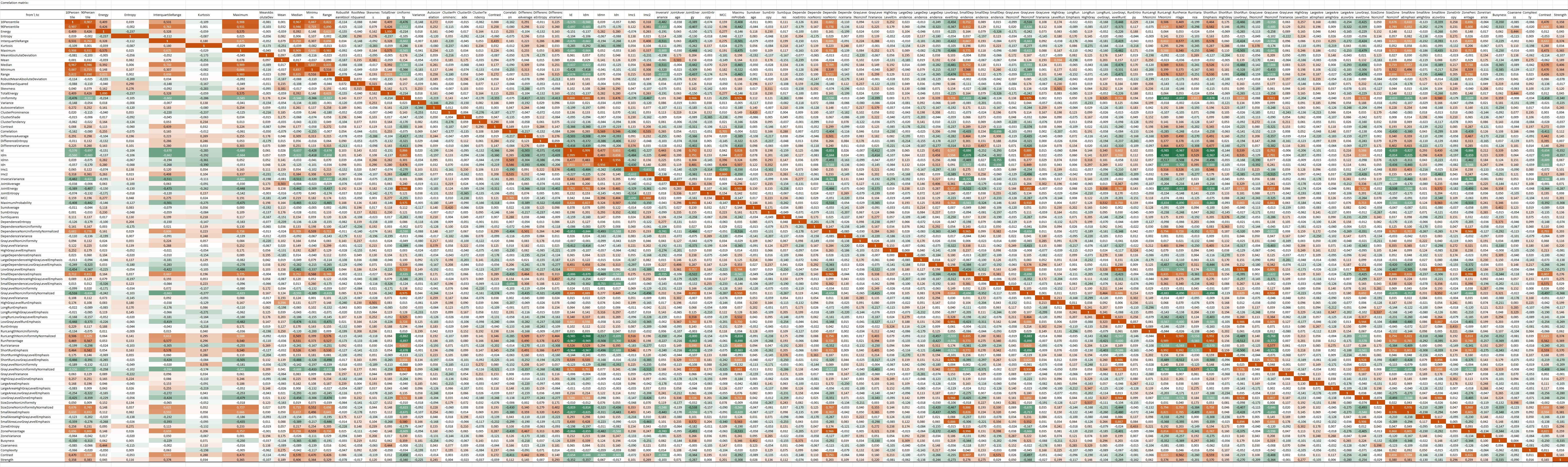

Supplement: Supplementary file 1 — Supplementary Figure 1: The correlation matrix of Least Absolute Shrinkage And SelectionOperator (LASSO). [file 11604_2026_1946_MOESM1_ESM.jpg]

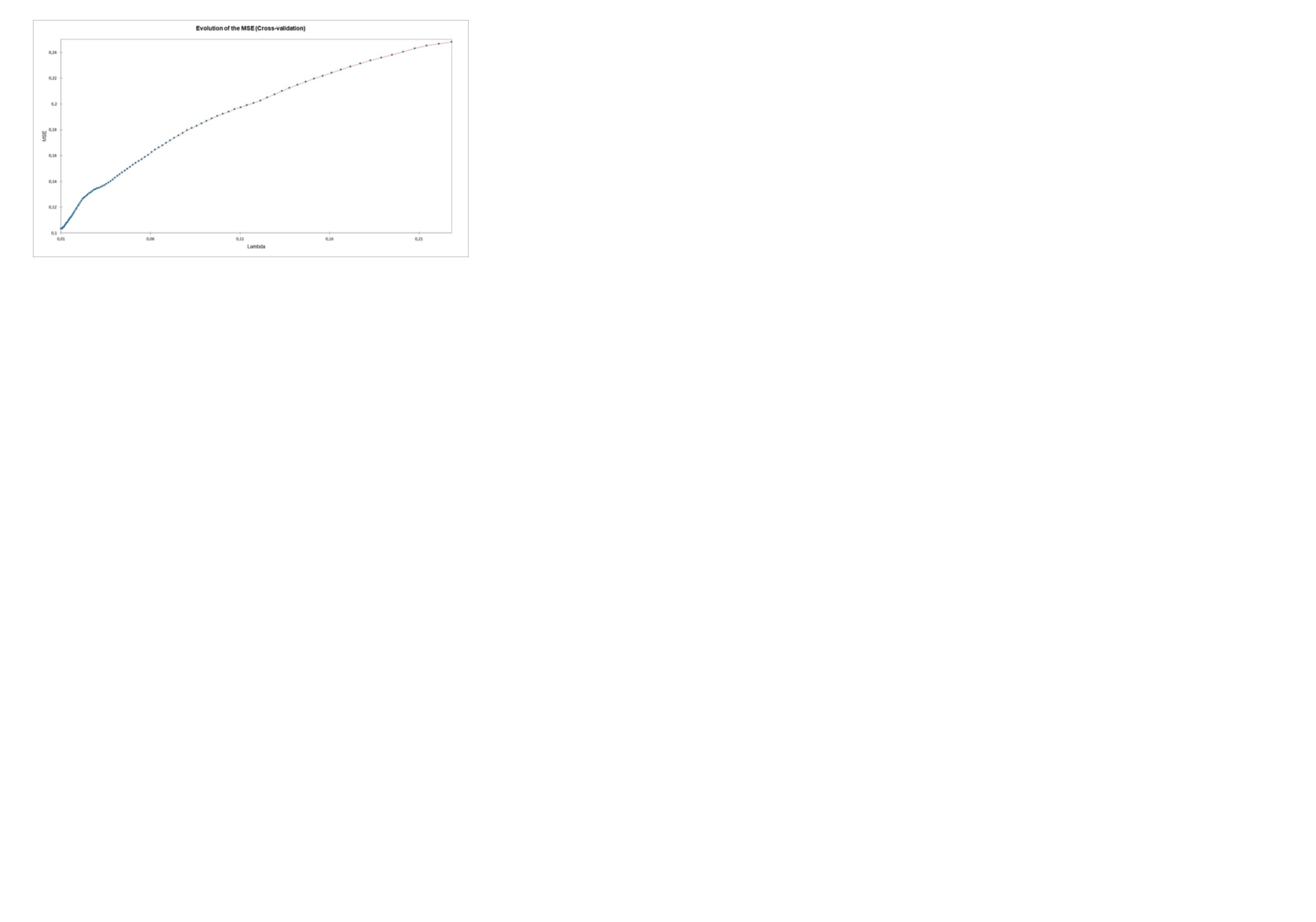

Supplement: Supplementary file 2 — Supplementary Figure 2: Correlation of mean standard error (MSE) with lambda parameter. [file 11604_2026_1946_MOESM2_ESM.png]
